# A Systematic Review about Cervical Pregnancy and our Experience

**DOI:** 10.15388/Amed.2024.31.1.13

**Published:** 2024-02-27

**Authors:** Konstantinos Nikolettos, Efthymios Oikonomou, Sonia Kotanidou, Nektaria Kritsotaki, Dimitrios Kyriakou, Panagiotis Tsikouras, Emmanouil Kontomanolis, Angeliki Gerede, Nikos Nikolettos

**Affiliations:** Obstetric and Gynecologic Clinic, Medical School, Democritus University of Thrace, Alexandroupolis, Greece

**Keywords:** ectopic pregnancy, cervical pregnancy, methotrexate, ektopinis nėštumas, gimdos kaklelio negimdinis nėštumas, metotreksatas

## Abstract

**Background:**

Cervical ectopic pregnancy is a relatively rare type of ectopic pregnancy and has no standardized guidelines for management.

**Methods:**

This systematic review is based on the collection of case reports, published in PubMed/MEDLINE about the resolution of ectopic cervical pregnancies over the last decade and the presentation of a case managed in our healthcare unit. Studies involving cervical pregnancy in the first trimester with the presence of a viable embryo and β-hCG in the serum below 100.000 mIU/mL were included, while heterotopic pregnancies were excluded

**Results:**

Nineteen articles reporting twenty-three case reports are demonstrated explicitly emphasizing on the management techniques. There is no established approach for the management of this type of ectopic pregnancy

**Conclusion:**

It is important to consider the conservative approaches as first-line treatment in all cases of cervical pregnancy preserving fertility. Minimally invasive methods are also described and preferred as second-line treatment, as reported in our literature review

## Abstract (it)

**Įvadas:**

Gimdos kaklelio negimdinis nėštumas yra palyginti retas negimdinio nėštumo tipas, kuriam nėra standartizuotų gydymo gairių

**Metodai:**

Ši sisteminė apžvalga paremta PubMed/MEDLINE publikuotų atvejų aprašymų apie negimdinio gimdos kaklelio nėštumo sprendimą per pastarąjį dešimtmetį rinkiniu ir mūsų sveikatos priežiūros skyriuje tvarkyto atvejo pristatymu. Buvo įtraukti tyrimai, susiję su gimdos kaklelio nėštumu pirmąjį trimestrą, kai yra gyvybingas embrionas ir β-hCG serume yra mažesnis nei 100 000 mIU/ml, o heterotopinis nėštumas nebuvo įtrauktas

**Rezultatai:**

Įvertinti devyniolika straipsnių, kuriuose pateikti dvidešimt trijų atvejų aprašymai, kuriuose aiškiai pabrėžiami gydymo metodai. Nėra nusistovėjusio šio tipo negimdinio nėštumo gydymo metodo

**Išvada:**

Svarbu, kad visais vaisingumą išsaugančio gimdos kaklelio nėštumo atvejais konservatyvūs metodai būtų laikomi pirmos eilės gydymo metodais. Minimaliai invaziniai metodai taip pat aprašyti ir jiems teikiama pirmenybė kaip antros eilės gydymui, kaip nurodyta mūsų literatūros apžvalgoje

## Introduction

Ectopic cervical pregnancy (CP) is the implantation of a gestational sac within the endocervical canal beneath the internal os, and is a rare form of nontubal ectopic pregnancy. The presence of a gestational sac below the internal os, surrounded by blood flow using Doppler and the absence of ‘sliding sign’ indicate the ultrasonography criteria of a CP [1,2]. This type of ectopic pregnancy occurs in less than 1% of all ectopic pregnancies and the incidence varies from one in 1,000 to one in 18,000 pregnancies [3–6].

Some of the main risk factors associated with CP are dilatation and curettage (D&C) or previous ectopic pregnancy, smoking, intrauterine adhesions, maternal age and assisted reproduction techniques [2,3].

Τhe traditional treatment of cervical pregnancy was initially hysterectomy, due to the excessive vaginal hemorrhage it may cause [2,7]. Over time, early diagnosis of ectopic pregnancies led to the introduction of new treatment approaches preserving fertility. Systemic or local injection of Methotrexate (MTX) and local injection of Potassium Chloride (KCL) are the main conservative methods described in the literature [8,9]. Invasive methods have also been presented, such as hysteroscopic or laparoscopic techniques with or without hemostatic management and laparoscopic uterine artery embolization (UAE) [10,11].

The purpose of this report is to demonstrate a case of CP that presented in our healthcare unit, and the effective treatment we have used, as well as to compare with similar case reports from the literature published in the last decade.

## Methods

This systematic review created according to the guidelines of the Preferred Reporting Items for Systematic Reviews and Meta-Analyses (PRISMA-P Statement). We used MEDLINE/PubMed for the collection of articles, published about the management of ectopic cervical pregnancies over the last decade (January 2013 – January 2023). For the search we used combinations of the following keywords and Medical Subject Heading (MeSH): ‘cervical,’ ‘ectopic’ and ‘pregnancy’.

Studies were included in the review if they reported a case of sonographically diagnosed cervical pregnancy in the first trimester presenting cardiac activity, written in English. The cut-point for the level of β-subunit of Human Chorionic Gonadotropin (β-hCG) was established at 100.000 mIU/mL. Case reports about heterotopic pregnancies, involving a gestational sac in cervical canal were excluded. Also, in case of missing or unclear information about the viability of the embryo or the level of the β-hCG, the article was excluded, as there was no contact with the authors. We additionally comprehended case reports related to our review, founded in the reference list of included studies.

## Study Selection

Using the search term ‘cervical pregnancy’ in MEDLINE/PubMed database we yielded 29.324 results. Among these results, many were irrelevant, so we used the term ‘ectopic cervical pregnancy’ to eliminate and specify the citations. This search yielded 1.544 articles. Using the filter ‘10 years,’ the citations diminished to 333 and by adding the filter ‘case reports’ we identified 126 reports.

The bibliography was processed by two authors (SK and NK) independently. Firstly, the articles related to ectopic cervical pregnancy were accumulated by title and abstract and then were analyzed furthermore for their eligibility. The articles that seemed to attain the inclusion criteria were studied for evaluation.

Limitations of this systematic review were the unavailability of adequate data in several case reports and the presence of relevant studies written in other languages than English. Articles that omitted to report the β-hCG level or presented a case missing information about the heart activity of the embryonic pole were excluded. Non-English articles were also excluded.

## Results

Initially, the first author selected by title and abstract 70 reports for assessment. Thirteen publications were excluded because of missing information about the viability of the embryo or the value of β-hCG, another thirteen studies were excluded due to presentation of heterotopic pregnancy, seven citations were excluded due to absence of cardiac activity and eighteen reports were omitted because the full text was not available in English. The second author selected 19 of these articles, that met the inclusion criteria for this systematic review, by examining the full text. Among these, two articles are presenting the management of two cases [12,13] and one of these is describing a series of three cases [14] .

**Figure 1:**
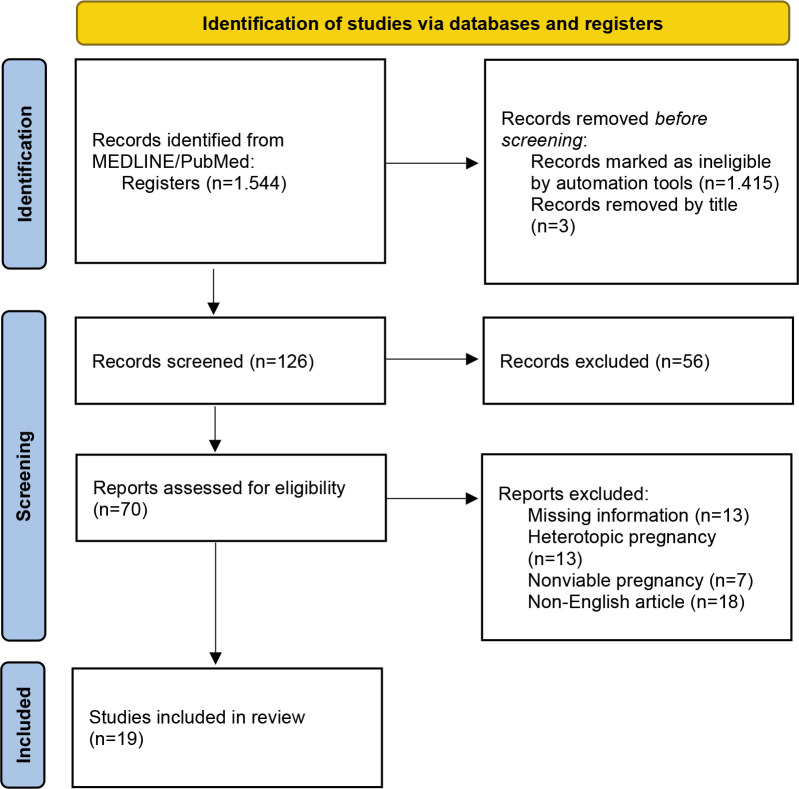
Flow diagram represents the study selection according to the guidelines of the Preferred Reporting Items for Systematic Reviews and Meta-Analyses (PRISMA-P Statement).

*Figure 1* presents the study selection of articles included in the systematic review.

The table below *(Table 1)* demonstrates the characteristics of the publications included analyzing the management in each case.

**Table 1:** Characteristics of the publications about cervical pregnancy included in this systematic review.

Author –year	Age	Obstetric History	Weeks of Gestation	Serum β-hCG (mIU/mL)	Conservative/ Invasive	Management	Outcome
**Alammari R. et al. 2017** **[15]**	39	G6P1 Ab4	7w1d	15.081	Invasive	Multidose systemic MTX + intra-amniotic 50mg MTX inj. + vaginal hysterectomy (patient’s choice)	Hysterectomy – Well recovered
**Bolaños-Bravo et al. 2019** **[16]**	30	G2P1	5w4d	16.189	Invasive	Multidose systemic MTX (60mg) + D&C + Foley catheter	Complete resolution
**Dziedzic J.M. et al. 2019** **[17]**	21	G1P0	4w3d	10.384	Conservative	Multidose MTX im (50mg/m^2^)	Complete resolution
**Guzowski G. et al. 2014** **[18]**	26	G1P0	6w	42.042	Invasive	Multidose systemic MTX (50mg/m^2^) + intra-amniotic KCl inj. + curettage	Complete resolution
**Han J.Y. et al. 2021** **[19]**	34	G1P0	6w	20.190	Conservative	Multidose MTX im (1mg/Kg) + fibrin sealant for hemostasis	Complete resolution
**Javedani Masroor M. et al. 2022** **[20]**	35	G1P0	37d	6.000	Conservative	4 doses MTX im (5mg/Kg) + intra-amniotic KCl inj.	Complete resolution
**Jiang J. et al. 2019** **[12]**	27	G3P1	6w2d	24.789	Invasive	HIFU + suction curettage	Complete resolution
**Jiang J. et al. 2019** **[12]**	29	G3P2	6w	32.506	Invasive	HIFU + suction curettage	Complete resolution
**Kumar N. et al. 2017** **[21]**	30	G1P0	5w6d	9.946	Conservative	Multidose MTX (1mg/Kg) + endocervical foley tamponade	Complete resolution
**Maglic R. et al. 2021** **[13]**	42	G3P2	7w1d	9.768	Invasive	Small-CaliberHysteroscopy	Complete resolution
**Maglic R. et al. 2021** **[13]**	37	G1P0	6w	13.737	Invasive	D&C	Complete resolution
**Mangino F.P. et al. 2019** **[22]**	41		6w6d	55.951	Invasive	5F bipolar electrode – cord section + resectoscopy	Complete resolution
**Mininni C. et al. 2021** **[23]**	43	G1P0	9w	85.220	Conservative	Single dose MTX + intra-amniotic KCl inj.	Massive vaginal bleeding followed by UAE with absorbable gelatin sponge
**Persadie R. J. et al. 2016** **[24]**	29	G3 Ab2	45d	14.689	Invasive	Multidose MTX (1mg/Kg) + removal with forceps and curettage	Complication by septicemia (E. coli)
**Petousis S. et al. 2015** **[25]**	41	G4P3	54d	28.590	Conservative	Single dose MTX (50mg/m^2^) + intra-amniotic KCl inj.	Complete resolution
**Saeng-anan U. et al. 2013** **[26]**	42	G2P0 Ab1	12w	60.826	Invasive	Intra-amniotic KCl inj. + systemic MTX + evacuation + curettage + balloon tamponade + abdominal hysterectomy	Hysterectomy
**Samal S. et al. 2015** **[27]**	26	G2P1	7w	74.014	Conservative	Intra-amniotic KCl inj. + multidose systemic MTX (1mg/Kg)	Complete resolution
**SpiezioSardo A. et al. 2017** **[28]**	36	G2P0 Ab1	5w5d	19.352	Invasive	Single dose im MTX (1mg/Kg) followed by intra-amniotic MTX inj. + 2 doses im MTX later + hysteroscopic resection of ectopic tissue	Complete resolution
**Takeda K. et al. 2018** **[29]**	44	G2P1	8w	71.964	Invasive	UAE + multidose systemic MTX (1mg/Kg)	Complete resolution
**Tanos V. et al. 2018** **[14]**	37	G2P1 Ec1	5w4d	1.650	Invasive	Vasopressin inj. – 5Fr scissors and hydro-dissection and resectoscope	Complete resolution
**Tanos V. et al. 2018** **[14]**	35	G1P0	6w	3.500	Invasive	Adrenalin inj. – 5Fr scissors and hydro-dissection	Complete resolution
**Tanos V. et al. 2018** **[14]**	30	G4Ab3	7w	13.790	Invasive	Vasopressin inj. – 5Fr scissors and hydro-dissection	Complete resolution
**Yeh C.Y. et al. 2022** **[30]**	31	G2P0 Ab1	6w	18.412	Invasive	Systemic MTX (50mg/m^2^) + hysteroscopic resection of ectopic tissue + foley catheter - postoperative day 1 MTX im (1mg/Kg)	Complete resolution

G: gravidity; P: parity; Ab: abortion; Ec: ectopic pregnancy; im: intramuscular; KCl: chloride potassium; inj: injection; MTX: methotrexate; HIFU: High-intensity focused ultrasound; D&C: dilation & curettage; UAE: uterine artery embolization

## Case report/presentation

A 37-year-old woman (G2P1) was referred to our department due to high suspicion of ectopic pregnancy. According to her last menstrual period, the gestational age was 7 weeks. Her medical history included a caesarian section 13 years ago and a miscarriage resolved by dilation and curettage (D&C) 6 months before the admission in our department.

The patient’s vital signs were stable and there was no sign of abdominal pain or vaginal bleeding during physical examination.

The initial routine blood tests, on admission in our department, revealed normal blood count. The level of hematocrit was 34.3%, hemoglobin was 11.7 g/dL and serum β-hCG level was 6.515 mIU/mL.

**Image 1:**
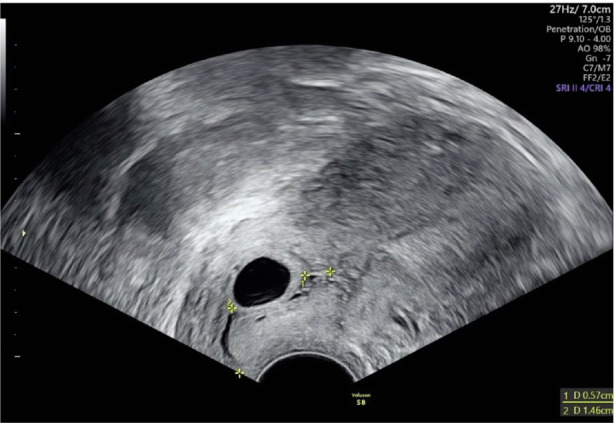
Gestational sac in the cervical canal.

**Image 2:**
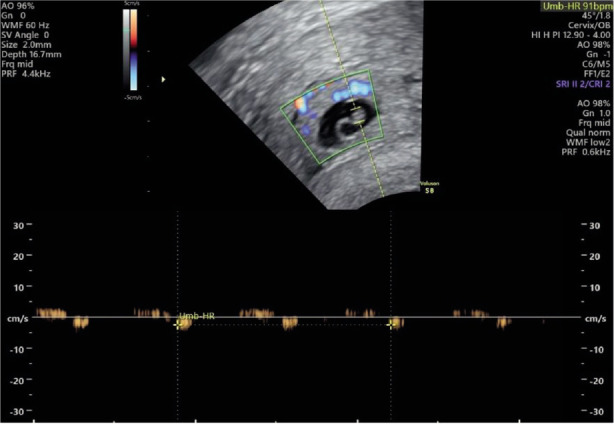
Presence of an embryonic pole with positive heart rate in the sac.

**Image 3:**
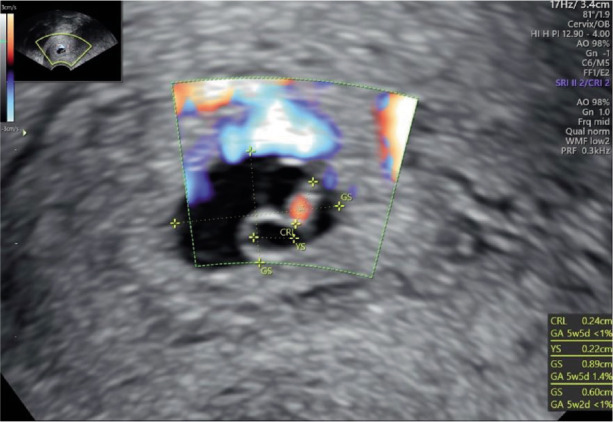
Doppler; blood flow surrounding gestational sac.

Transvaginal ultrasonography (TVUS) confirmed the presence of a gestational sac (0.89 cm x 0.60 cm) located in the cervical canal including a fetal pole with cardiac activity *(Images 1–3)*.

We decided to proceed to the management of the ectopic cervical pregnancy by administrating 75 mg methotrexate intramuscularly (IM) (50 mg/m^2^), repeated on days 3, 5 and 7.

**Image 4:**
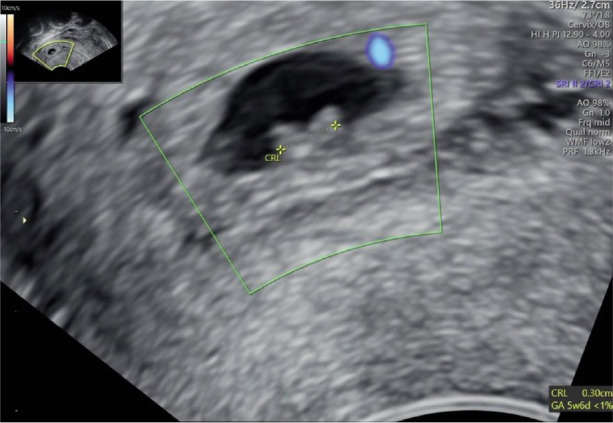
Ultrasound imaging on the 10th day presenting an embryonic pole with absence of heart activity.

On the 10th day of her admission, the ultrasound scan presented negative fetal heart rate and the level of serum β-hCG was 6.360 mIU/mL with stable hematocrit and hemoglobin level *(Image 4)*. On the 18th day of her admission to the hospital, the patient experienced massive vaginal bleeding, and she was transferred to the operating room. Suction and curettage were performed and the bleeding from the cervical pregnancy was controlled by placing two ligation sutures on the descending branches of the uterine artery.

Completing the invasive treatment for the cervical pregnancy, the β-hCG level was diminished to 978 mIU/mL, hematocrit level was 30.2% and hemoglobin 10 g/d and blood transfusion was not required and the patient discharged. The follow-up ultrasound scan on day 7 after discharge, showed a normal cervix and the β-hCG level was 40 mIU/mL.

## Discussion

In this systematic review, we evaluated a series of cases of ectopic cervical pregnancy and analyzed the obstetric history, gestational age, serum β-hCG levels, and management strategies employed in each case. The outcomes of these interventions were also assessed.

We found no established method or standardized guidelines for the management of ectopic cervical pregnancy. The management approach for ectopic cervical pregnancy varies depending on factors such as the timing and manner of presentation (e.g., asymptomatic or acute, early or delayed), the healthcare facility’s setting, and the expertise of the physician involved.

In the past thirty years, a range of methods has been employed for the conservative, nonsurgical treatment of cervical pregnancy [31].

Dziedzic et al. described the successful outcome of a case involving a primigravida with cervical ectopic pregnancy who received multiple doses of methotrexate (50 mg/m^2^) intramuscular, while Han et al. described a case in which the patient was referred to medical attention because of experiencing vaginal spotting and discomfort in the lower abdomen and underwent a treatment plan involving multiple doses of MTX, and the hemorrhage was effectively managed through the application of hemostatic agents (fibrin sealant) directly at the cervical bleeding site [17,19].

The administration of a single dose of methotrexate followed by KCL injection was reported in two cases by Petousis et al. and Mininni et al. In the first case, a successful outcome was achieved, while the second case was associated with massive vaginal bleeding three months later and embolization of the uterine artery using gelatin sponge [23,25].

The successful outcome of administering ultrasound-guided intra-amniotic potassium chloride followed by multiple doses of intramuscular methotrexate was described by Samal et al., while a modified method of administration of multiple intramuscular doses of methotrexate followed by intra-amniotic KCL injection was reported by Javedani Masrooret et al. [20,27].

Kumar et al. described the administration of multiple doses of methotrexate followed by the emergency placement of an endocervical Foley tamponade due to significant vaginal bleeding, a combination of a medical and a conservative compression procedure [21]. The implementation of an inflated Foley catheter has been demonstrated as an effective approach for managing cervical bleeding in cases of cervical ectopic pregnancies. Previous studies have shown that this method successfully achieves hemostasis in 92.3% of cases [32–35].

Small-Caliper hysteroscopy as a single treatment method with a successful outcome was reported by Maglic et al. [13]. Tanos et al. documented a series of three cases in which hysteroscopy was employed as the exclusive therapeutic approach. They utilized a 2.8 mm hysteroscope with a 5Fr working channel, administered either adrenaline or vasopressin in an avascular area surrounding the sac, and achieved detachment of the pregnancy sac using 5Fr scissors and hydrodissection. In one case, resectoscopy was performed due to partial detachment of the sac during hysteroscopy [14]. Mangino et al. combined hysteroscopy with resectoscopy in a single case, where they performed the opening of the sac and removal of the umbilical cord using the former technique, and subsequently extracted the sac using the latter technique [22]. Yeh et al. performed hysteroscopic resection of ectopic tissue with subsequent administration of an intramuscular dose of methotrexate to achieve the desired outcome [30].

Evacuation and curettage, with or without cervical cerclage, have been reported as effective conservative surgical treatments for cervical pregnancy. This case report of ectopic cervical pregnancy mentions the administration of multiple doses of methotrexate (4 doses, 50 mg/m^2^) and underwent suction curettage followed by close monitoring and placement of hemostatic sutures in the branches of the uterine artery due to sudden vaginal bleeding. Comparable effective management was documented by Mahdavi et al. who described the placement of cerclage sutures to encircle the cervical canal, followed by curettage. Following this, the McDonald cerclage suture was tightly tied [36].

Dilation and curettage was used by Maglic et al., and a modified approach of performing high-intensity focused ultrasound (HIFU) a prophylactic procedure before suction curettage was described by Jiang et al. [12,13]. The procedure of dilation and curettage (D&C) performed in isolation poses a 40% probability of necessitating a subsequent hysterectomy [37]. Persadie et al. reported the administration of multiple doses of methotrexate followed by removal with forceps and curettage, a therapeutic method that resulted in septicemia caused by E. Coli [24]. Bolaños-Bravo et al. mentioned a more sophisticated approach in which they combined the systematic administration of methotrexate followed by dilation and curettage, and subsequently the placement of a Foley catheter, to prevent any potential urgent hemorrhage [16].

Invasive methods have also been described.

Takeda et al. described the use of bilateral uterine artery embolization followed by intramuscular administration of methotrexate on the first postoperative day [29]. The risk factors associated with the recurrence of vaginal bleeding following uterine artery embolization (UAE) include the presence of fetal cardiac activity prior to therapy, persistent elevated levels of human chorionic gonadotropin (hCG), and the reappearance of blood flow signals around the gestational sac located within the cervix [10].

Hysterectomy, whether abdominal or vaginal, is considered the last and less preferable option, and it is used in cases where other methods have failed or have complications such as severe hemorrhage.

Alammari et al. presented a case study of a 42-year-old patient in which the combined therapy involving intra-amniotic injection of 50 mg of MTX under ultrasonographic guidance and subsequent administration of a multidose-regimen of MTX with folinic acid rescue proved ineffective, leading to the ultimate treatment option of vaginal hysterectomy. The patient preferred the aforementioned treatment after considering various alternatives such as UAE followed by D&C and repeated intra-amniotic injections with MTX or other agents as she had completed her family planning [15]. Abdominal hysterectomy has been described by Saeng-anan U. et al. as the definitive treatment in a patient with an ectopic cervical pregnancy, in which conservative therapy involving intrafetal potassium chloride (KCL) injection was infused under ultrasound guidance and the administration of multiple doses of MTX resulted in the termination of fetal cardiac activity. However, this approach led to massive vaginal hemorrhage, hypovolemic shock, and unstable vital signs, despite the application of Bakri SOS balloon tamponade. Postoperatively, the patient experienced disseminated intravascular coagulation and acute tubular necrosis due to the significant blood loss [26]. Total abdominal hysterectomy is considered the optimal therapeutic intervention for women with cervical pregnancies detected in the second trimester, exhibiting unstable vital signs, experiencing excessive vaginal bleeding, presenting with concomitant uterine pathology, belonging to the Jehovah’s Witnesses faith, and having fulfilled their desire for offspring [10].

Systemic administration of methotrexate is the most commonly employed conservative treatment method for cervical pregnancy, with a success rate of 91%. However, a cervical ectopic pregnancy characterized by a serum β-hCG level equal to or exceeding 10.000 mIU/mL, a gestational age of at least 9 weeks, embryonic cardiac activity, and a crown-rump length greater than 10 mm was found to have a higher likelihood of unsuccessful outcomes when treated primarily with methotrexate [38].

The main limitation of conservative management for cervical pregnancy is the risk of acute complications leading to hemorrhage. Therefore, nonsurgical approaches should only be carried out in specialized medical facilities with access to immediate medical attention [39].

As far as more invasive options are concerned, both high-intensity focused ultrasound (HIFU) and uterine artery embolization (UAE) in combination with hysteroscopic curettage are safe and efficacious in the treatment of patients with cervical pregnancy (CP). In comparison to UAE, HIFU emerges as a preferable and more effective treatment option due to its less invasive nature, shorter interval time, reduced duration of hospitalization, and quicker recovery time for menstruation, lower incidence of adverse reactions, and fewer postoperative complications [40].

Primary hysterectomy may remain the favored treatment approach in cases of uncontrollable bleeding, diagnosis of cervical pregnancy in the second or third trimester of pregnancy, and potentially as a means to prevent the need for emergency surgery and blood transfusion in a woman who does not wish to preserve fertility [41].

## Conclusion

Overall, the reported cases in this review highlight the lack of standardized guidelines for the management of ectopic cervical pregnancy. The varying approaches employed in different cases reflect the individualized nature of treatment decisions, which depend on factors such as the patient’s presentation, physician expertise, and available resources. Without a doubt, it is essential to consider conservative approaches as the first line of treatment in all instances of cervical pregnancy, including in women who have finished their childbearing years. The administration of methotrexate in multiple doses, in combination with intra-amniotic potassium chloride (KCL) infusion, appears to be the first-line therapeutic choice especially for primigravid women. Further research and consensus in the medical community are warranted to develop standardized protocols and guidelines to optimize the management of ectopic cervical pregnancy and improve patient outcomes.
